# Synthesis of Activated Carbon from *Trachycarpus fortunei* Seeds for the Removal of Cationic and Anionic Dyes

**DOI:** 10.3390/ma15061986

**Published:** 2022-03-08

**Authors:** Esraa M. Bakhsh, Muhammad Bilal, Maqsood Ali, Javed Ali, Abdul Wahab, Kalsoom Akhtar, Taghreed M. Fagieh, Ekram Y. Danish, Abdullah M. Asiri, Sher Bahadar Khan

**Affiliations:** 1 Department of Chemistry, Faculty of Science, King Abdulaziz University, P.O. Box 80203, Jeddah 21589, Saudi Arabia; ibakhsh@kau.edu.sa (E.M.B.); kaskhan@kau.edu.sa (K.A.); tfagieh@kau.edu.sa (T.M.F.); eydanish@kau.edu.sa (E.Y.D.); aasiri2@kau.edu.sa (A.M.A.); 2 Department of Chemistry, Kohat University of Science & Technology, Kohat 26000, Khyber Pakhtunkhwa, Pakistan; maqsoodmwt@gmail.com (M.A.); javidali@kust.edu.pk (J.A.); 3 Department of Pharmacy, Kohat University of Science & Technology, Kohat 26000, Khyber Pakhtunkhwa, Pakistan; wahabscholar@yahoo.vom; 4 Center of Excellence for Advanced Materials Research, King Abdulaziz University, P.O. Box 80203, Jeddah 21589, Saudi Arabia

**Keywords:** *Trachycarpus fortunei* seeds, activated carbon, adsorbent, dyes, water purification

## Abstract

The removal of dyes from industrial effluents is one of the most important industrial processes that is currently on academic demand. In this project, for the first time, *Trachycarpus fortunei* seeds are used as biosources for the synthesis of activated carbon (AC) using physical as well as acid–base chemical methods. The synthesized AC was initially characterized by different instrumental techniques, such as FTIR, BET isotherm, SEM, EDX and XRD. Then, the prepared activated carbon was used as an economical adsorbent for the removal of xylenol orange and thymol blue from an aqueous solution. Furthermore, the effect of different parameters, i.e., concentration of dye, contact time, pH, adsorbent amount, temperature, adsorbent size and agitation speed, were investigated in batch experiments at room temperature. The analysis of different techniques concluded that the pyrolysis method created a significant change in the chemical composition of the prepared AC and the acid-treated AC offered a high carbon/oxygen composite, which is graphitic in nature. The removal of both dyes (xylenol orange and thymol blue) was increased with the increase in the dye’s initial concentration. Isothermal data suggested that the adsorption of both dyes follows the Langmuir model compared to the Freundlich model. The equilibrium time for AC biomass to achieve the removal of xylenol orange and thymol blue dyes was determined to be 60 min, and the kinetic data suggested that the adsorption of both dyes obeyed the pseudo-second order model. The optimal pH for thymol blue adsorption was pH 6, while it was pH 2 for xylenol orange. The adsorption of both dyes increased with the increase in the temperature. The influence of the adsorbent amount indicated that the adsorption capacity (mg/g) of both dyes reduced with the rise in the adsorbent amount. Thus, the current study suggests that AC prepared by an acid treatment from *Trachycarpus fortunei* seeds is a good, alternative, cost effective, and eco-friendly adsorbent for the effective removal of dyes from polluted water.

## 1. Introduction

The supervision and treatment of wastewater sources is a field that has demanded great technical awareness for decades [1]. Nowadays, the industrial revolution leads to consuming high amounts of water along with organic-based chemicals. Due to the strict limitations implemented on the organic content of industrial effluents, there have been extensive efforts to develop efficient remediation technologies for wastewater treatment. Synthetic dyes are considered as a main category of chemicals that are extensively employed in industries for the coloration of products, especially textiles. Such dyes are ultimately discharged into effluents, thus influencing aquatic environments [2,3]. The quality of water is highly influenced by the color of the discharged dyes because the presence of trace concentrations of dyes in water even by mg/L is visible, which is unfavorable [4]. It is well known that dyes are the main type of contamination in wastewater, which are released in water reservoirs that absorb and reflect sunlight and, ultimately, disturb aquatic life and affect the food chain [5]. In addition, most of such dyes are carcinogenic in nature, thus posing a serious threat to aquatic organisms [6]. The main structure of synthetic dyes is aromatic rings that become highly stable by opposing the departure upon exposure to sweat, soap, water, light and oxidizing agents [7]. As a result, the dyes are not predictable for natural behavior, especially for aerobic digestion as well as for oxidizing agents [8]. However, reactive dyes are soluble in water and 10–50% of dyes remain in waters leading to strongly colored effluents that cause serious problems in the environment [9]. Thus, industrial pollutants, especially textile dyes, are highly hazardous and potentially carcinogenic causing environmental degradation along with diverse diseases to humans and other organisms. They decompose to carcinogenic aromatic amines under anaerobic conditions, which ultimately affect the structure and functionality of the ecosystem. The main side effects of dyes may involve mutations, cancer and irritations of eye and skin. 

In general, conventional remediation techniques are of a physical, chemical and biological nature including ion exchange, adsorption [10], oxidation processes [11,12], catalytic reduction [13,14], membrane filtration [15,16], coagulation/flocculation, ozonation, electroflotation, electrokinetic coagulation, electrochemical destruction, irradiation, precipitation and biotreatments [17]. Such techniques may be efficient in the purification of effluents, however, most of them are expensive and technically complicated [18]. Adsorption is a physical adherence or bonding of ions or molecules present on the surface of another molecule. It has many advantageous features, such as effectiveness, ease of operation, simple recovery and the ability to reprocess the adsorptive material [19]. In this treatment, the dye molecules are removed by attaching dyes to the surface of the asbestos. This connection is caused by physical or chemical forces between the surface and the molecules or ions. Since the compression is the exterior occurrence, it will strongly depend on the specific float-up area or the space available for the dye molecules to access the surface of the adsorbent material. 

Several studies have been conducted using AC. However, it is recognized that the use of industrial AC used for dye elimination is very expensive; therefore, research was conducted to find agricultural, industrial or urban waste to produce AC or to use raw materials as adsorbents [20]. Commercial systems mostly use AC as adsorbents to eliminate dyes from wastewater and provide a superb adsorption potential [21]. Several methods have been explored to extend the search to cheaper and more useful adsorbents. In spite of the multiplicity of adsorbents, it has been established that AC is still useful to remove contaminated water from plants and is still effective in a gaseous situation. Activated carbon (AC) is a well-known adsorbent that is employed in industrial processes and has a micro porous homogenous structure with a high surface area. However, the limitation of implementing AC is due to its high cost and difficulty to regenerate [22]. Therefore, researchers have paid attention to the production of AC from renewable sources using low-cost and local agricultural waste. Several underused wastes that have been employed to explore possible sources for AC are forests, bagasse [23], coconut peat [24], corncobs [25], cassava shed [26], tobacco stems [27], hazelnut [28], and some other. 

The fruit of *Trachycarpus fortunei* is used in making polishes, wax papers and carbon papers, and the seeds are usually discarded, but have potential to be used in the removal of dyes from industrial effluents because they are very hard and have high carbon constituents. Therefore, in this present study, *Trachycarpus fortunei* seeds were used as biomass for the synthesis of activated carbon. This prepared AC was then used as an adsorbent for the removal of xylenol orange and thymol blue dyes from aqueous solutions.

## 2. Experimental Procedure

### 2.1. Chemicals/Solvents

The chemicals utilized in the project were of analytical grade and used as obtained from the suppliers. Xylenol orange (XO), thymol blue (TB), hydrochloric acid (HCl), phosphoric acid (H_3_PO_4_), and sodium hydroxide (NaOH) were purchased from Sigma-Aldrich, Zama Zam Supermarket, Karachi, Pakistan. Deionized water was employed in the preparation of the solutions. XO, which is a cationic dye, and TB, an anionic dye, were selected as adsorbates. Raw biomass and AC treated with acid and base were prepared from T. F. seeds, which were selected as adsorbent.

### 2.2. Preparation of the Adsorbent

#### 2.2.1. Raw Biomass

T. F. seeds were collected from the local areas of Lakki Marwat, Khyber Pakhtunkhwa, Pakistan, in June–July 2018. The seeds were washed with deionized water for removing dust particles. Then, the seeds were dried for a couple of days under sunshine. The dried seeds were then ground to powder form by using a grinder and then sieved to acquire a homogenous adsorbent with a known particle size (with the ranges of 45–90 µm and 90–212 µm).

#### 2.2.2. Activated Carbon (AC)

AC was prepared by heating T. F. seeds at 550 °C in a nitrogen atmosphere for 30 min. The prepared composite was activated with H_3_PO_4_ and NaOH using the ratio of 1:5 and then washed several times with deionized water. The prepared composite was dried at 110 °C in an oven for two hours to remove the moisture contents from the sample. The formation of both raw biomass and AC was confirmed by using different characterization techniques, such as FTIR, XRD, SEM/EDX, and BET.

### 2.3. Instrumentation

The following instruments were used for the characterization of AC and adsorption analysis. FTIR analysis of the biomass and AC was carried out by using a FTIR Spectrophotometer (Spectrum Two, Perkin Elmer, Waltham, MA, USA) in the range of 400–4000 cm^−1^. The surface area and porosity of biomass and AC were characterized by NOVA 2200e, Quantachrome, Ashland, Virginia, USA., by adsorption–desorption isotherms of nitrogen at −196 °C. For each analysis, 0.3 g of the sample was used. SEM images of biomass and AC were studied by scanning electron microscope (SEM) (JSM-5910, JEOL, Tokyo, Japan). The elemental analysis of the biomass and AC was performed by using Energy Dispersive Spectroscope (INCA 200, Oxford Instruments, Oxford, UK) with SEM (JSM-5910, JEOL, Tokyo, Japan). The crystal structures of the biomass and AC were investigated by X-ray diffractometer (JDX-3532, JEOL, Tokyo, Japan) using Cu Kα (λ = 1.5418 Å). The adsorption capacity of both the biomass and AC was determined by UV-Vis Spectrophotometer (Shimadzu UV-1800, Shimadzu Corporation, Tokyo, Japan). The instrumental conditions were: wavelength range: 190–800 nm; scan rate: 150–600 nm min^−1^; and slit: 1.0 nm.

### 2.4. Adsorption

#### Preparation of Stock Solutions

The basic cationic dye (XO) and the anionic dye (TB) were taken as adsorbates and 1000 mg/L stock solution was prepared for both dyes, separately, by dissolving 1 g of each dye in 1000 mL volumetric flasks and diluted up to the mark by using deionized water. Further solutions of different concentrations (mg/L) were prepared from these stock solutions by using the dilution method.

### 2.5. Adsorption of TB and XO on Biomass and AC Treated with Acid and Base

The influence of various parameters, i.e., initial dye concentration, contact time, pH, adsorbent amount, temperature, adsorbent size and rpm, on the adsorption of XO and TB on biomass and AC were investigated as follows:

#### 2.5.1. Dye Concentration

To explore the influence of dye concentration, 50 mL of various concentrations of both dyes (1 mg/L to 1000 mg/L) were prepared separately, and 0.1 g of adsorbent was added to each solution. All solutions were stirred on a water bath at a rate of 140 rpm for 60 min. After 60 min of stirring, all solutions were filtered. The filtrate was transferred to sample bottles for UV analysis. 

#### 2.5.2. Contact Time

A total of 50 mL of both dyes’ solutions (100 mg/L TB and 50 mg/L XO) were taken in different conical flasks to study the contact time influence on TB and XO dye adsorption on biomass and AC. An amount of 0.1 g of adsorbent was mixed with each solution. All solutions were stirred at different intervals of time (1, 5, 10, 20, 30, 60, 120 and 180 min) on a water bath shaker with continuous stirring at 140 rpm speed. After stirring, all solutions were filtered, and the filtrate was transferred to sample bottles for UV analysis.

#### 2.5.3. pH

Different solutions (50 mL) of TB and XO (100 mg/L TB and 50 mg/L XO) were placed in separate conical flasks. The pH of each solution was adjusted (pH 2–14) with 0.1 M HCl and 0.1 M NaOH solution. A 0.1 g of biomass and AC adsorbents was individually mixed with each solution and all solutions were stirred on a water bath shaker for 60 min at 140 rpm. Then, all solutions were filtered by Whatman filter paper, and the filtrate was transferred to sample bottles for UV analysis.

#### 2.5.4. Temperature

Different solutions (50 mL) of 100 mg/L TB and 50 mg/L XO were prepared in separate conical flasks and 0.1 g of biomass and AC adsorbents was added separately to each solution. All solutions were shaken on a water bath shaker for 60 min at different temperatures (10–80 °C) at the constant speed of 140 rpm. After shaking, all solutions were filtered, and the filtrate was transferred to sample bottles for UV analysis.

#### 2.5.5. Adsorbent Amount

A total of 50 mL of dyes solutions having a concentration of 100 mg/L TB and 50 mg/L XO were placed in separate conical flasks and different amounts of adsorbent (0.1, 0.2, 0.3, 0.4 and 0.5 g) were mixed with each solution separately. All the solutions were shaken for 60 min at 140 rpm. After shaking, all solutions were filtered, and the filtrate was transferred to sample bottles for UV analysis.

#### 2.5.6. Adsorbent Size

A total of 50 mL of different solutions of 100 mg/L TB and 50 mg/L XO were placed in separate conical flasks. A total of 0.1 g of adsorbent of different particle sizes (below 45 µm, 45–90 µm and 90–212 µm) was added to each solution. All solutions were shaken on a water bath shaker for 60 min at the rate of 140 rpm. After shaking, each solution was filtered, and the filtrate was transferred to sample bottles for UV analysis.

#### 2.5.7. RPM: Shaker Speed

A total of 50 mL of different solutions of 100 mg/L TB and 50 mg/L XO were taken in separate conical flasks and 0.1 g of biomass and AC adsorbents was added separately to each solution. All solutions were shaken on a water bath shaker with different speeds (35, 70, and 140 rpm) for 60 min. After shaking, all solutions were filtered, and the filtrate was transferred to sample bottles for UV analysis.

## 3. Results and Discussion

### 3.1. Characterization

#### 3.1.1. FT-IR

**Figure 1:**
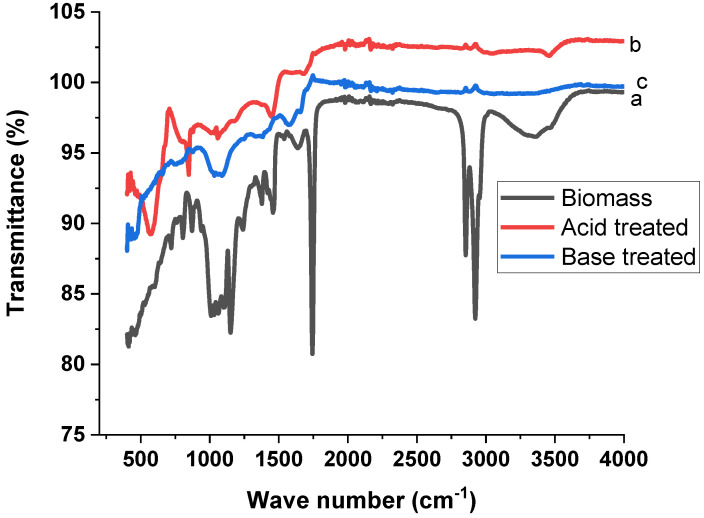
FTIR spectra of (**a**) biomass, (**b**) acid-treated AC and (**c**) base-treated AC.

The moiety of biomass (T. F. seeds) and AC derived from biomass as obtained by FTIR analysis, as displayed in Figure 1. Different functional groups were obtained from the data of biomass, which includes hydroxyls, amines, unsaturated hydrocarbons, aldehydes, and carbonyl compounds. Nevertheless, pyrolysis led to losing some such peaks in the AC.

In the biomass, the noticed peak at 3358 cm^−1^ is ascribed to the O–H stretching [29]. The asymmetric and symmetric stretching of the methylene (C–H) group was observed at 2923 cm^−1^ and 2854 cm^−1^, respectively [30]. The peak observed at 1743 cm^−1^ is owed to the C=O stretching [31]. The peaks observed at 1457 cm^−1^ are due to the C–H bending and at 1377 cm^−1^ is due to the N–O stretching vibration [29]. C–O stretching vibrations appeared at 1239 cm^−1^ [32]. The peaks observed at 1150 cm^−1^ and 1638 cm^−1^ are due to the C–O stretching vibrations and C=C stretching, respectively [29].

Most of the peaks disappeared in the FTIR spectra of the AC treated with acid and base. Some new peaks appeared in the case of the AC treated with acid. The peaks displayed at 876 cm^−1^ and 1574 cm^−1^ are owed to the functional groups of aromatic C–H bonds [33] and C=C stretching [32], respectively. In the case of AC treated with base, the peak observed at 1057 cm^−1^ is ascribed to C–O stretching [34]. C–H bending appeared at 1449 cm^−1^ [35]. The peak obtained at 1683 cm^−1^ is related to C=O stretching [36]. These results indicate that the pyrolysis process caused a significant change in the chemical composition. In both acid- and base-treated AC, the peaks for OH, CH and C=O disappeared, showing that the heat treatment removed these moieties from biomass and the polyaromatic type AC was formed. The formation of AC is also supported by appearance of C=C bending at 847 cm^−1^ and at 580 cm^−1^.

#### 3.1.2. BET Analysis

To determine the surface area of biomass and AC treated with acid and base, characterization was performed by N_2_ adsorption–desorption isotherm analysis at −196 °C. For each analysis, a sample of 0.3 g was used. The specific surface area was assessed by the BET method and the results obtained are illustrated in Table 1. The table shows that, in both acid- and base-treated AC samples, the surfaces were increased. This increase in surface area is also supported by the SEM results, as shown later. However, due to limitations (low value of C, i.e., 10), the BET results did not show a clear picture of the increase in the surface area of the prepared AC. The high increase in surface area was also observed in the acid-treated AC in the previous studies [37].

#### 3.1.3. SEM

**Figure 2:**
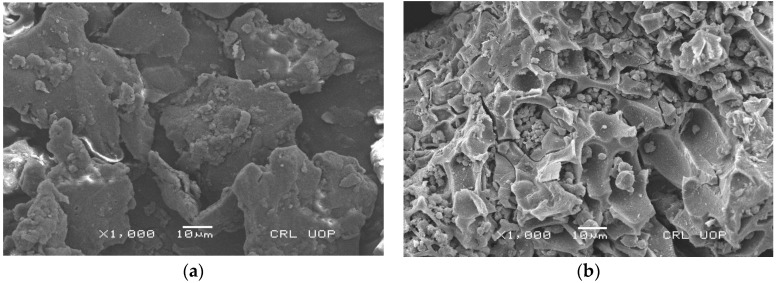

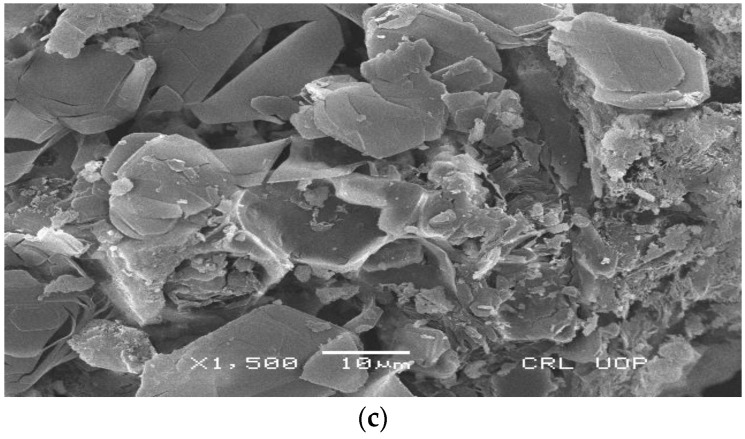
SEM images of (**a**) biomass, (**b**) acid-treated AC and (**c**) base-treated AC.

SEM was employed to evaluate the morphology of biomass and AC treated with acid and base samples as well as the important changes in the morphology of the prepared samples. The results given in Figure 2 show the SEM images of biomass (a), AC treated with acid (b) and AC treated with base (c). These results specify that the surface morphology of biomass is smooth and different residues were deposited, while no apparent pores are observed. The synthesis of AC treated by acid produced significant pores in the surface. Additionally, the disintegration of the surface took place (Figure 2b). The treatment of AC with base also produced a significant change in the morphology of the biomass. However, the base treatment produced only the disintegration of the surface, while no apparent pores were produced.It has been reported that acid- and base-treated ACs were compared and found that acid-treated AC showed the best results [38]. These results indicate that acid-treated AC has a high porous structure, which is one of the main desirable outcomes of the adsorption process.

#### 3.1.4. EDX

**Figure 3:**
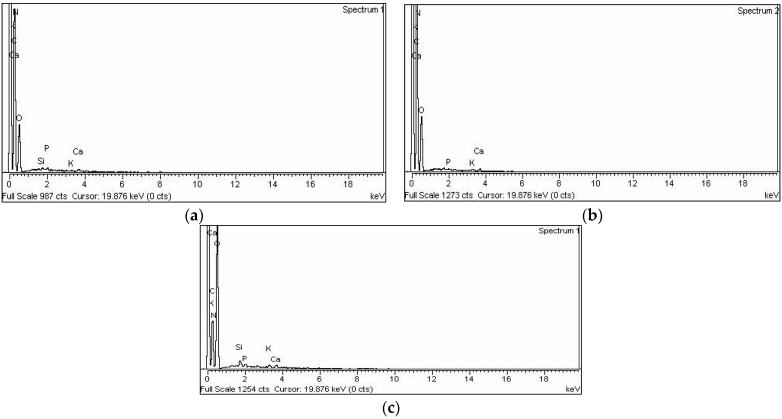
EDX spectra of (**a**) biomass (**b**), acid-treated AC and (**c**) base-treated AC.

EDX spectra of biomass and AC treated with acid and base are shown in Figure 3, and the data obtained are tabulated in Table 2. The samples with the maximum amount of carbon and the slightest amount of oxygen are held to be the most efficient ACs because they have a higher surface area and graphitic-type structure. Regarding Table 2, in the biomass sample, it is observed that the amount of carbon and oxygen are 64.33% and 25.78% by atom, respectively. On the other hand, the acid-treated AC contains 83.24% and 14.26% of carbon and oxygen, respectively. Similarly, base-treated AC contains 56.21% and 30.08% of carbon and oxygen, respectively. Table 2 also shows that the amount of nitrogen in biomass is 9.98% (by weight), while it almost disappeared (0.18% by weight) in the acid-treated AC, but it increased to 12.01% by weight in the base-treated AC. These results demonstrate that the acid treatment caused a significant change in the elemental composition of the prepared AC. The high carbon/oxygen ratio in the acid-treated AC is supported by the SEM (Figure 2) and BET (Table 1) results and are in line with previous investigations [39].

In addition to the aforementioned elements, EDX analysis also showed the presence of Si, P, K, and Ca in biomass, which slightly change in the AC treated with acid and base.

#### 3.1.5. Powder XRD

**Figure 4:**
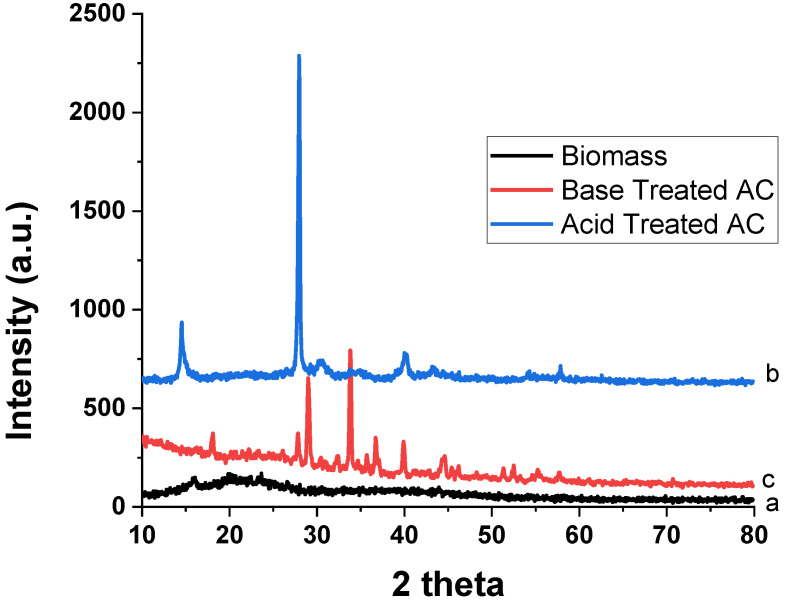
Powder XRD patterns of (**a**) biomass, (**b**) acid-treated AC and (**c**) base-treated AC.

Powder XRD analysis was carried out to determine the crystal nature of biomass and AC treated with acid and base, following the procedure discussed in Section 2.3 (Figure 4). The figure illustrates that the powder XRD patterns of biomass showed a broad diffraction peak at two theta positions of 23°, which demonstrates that the biomass sample is amorphous with small crystallinity. The amorphous nature of biomass is also supported by our previous studies carried out on the seeds of *Datura metel* [40]. The synthesis of AC from the obtained *Trachycarpus fortunei* seeds showed a crystalline structure. The crystalline nature is characterized by a peak at two theta positions of 27°. However, the crystallinity of the synthesized AC is slightly different according to the different chemical treatments. In the case of the AC treated with acid, this peak appears at two theta positions of 27°. This confirms the formation of AC, while in the case of the base-treated AC, the morphology totally changed. Shifted peaks occurred towards the high two theta positions, i.e., at 29°. Additionally, a peak at two theta positions of 35° appeared, which is not recognized. These results indicate that the chemical treatment of AC produced a significant change in the produced AC and the acid-treated AC produced a graphitic-type AC [41].

### 3.2. Adsorption of XO and TB on Biomass and AC Treated with Acid and Base

Adsorption experiments were performed by taking varied concentrations of primary dyes, contact times, pH, adsorbent concentration, temperature, adsorbent size and RPM to determine the uptake capacity of the organized seeds and ACs.

#### 3.2.1. Dye Concentration

**Figure 5:**
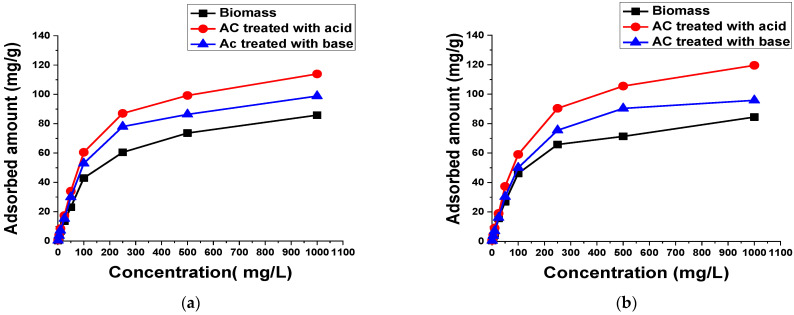
Effect of (**a**) TB and (**b**) XO concentration on the adsorption of 50 mL solution using 0.1 g biomass and AC treated with acid and base for 60 min at 140 rpm and room temperature.

The influence of dye concentration on the adsorption of TB and XO was studied to evaluate the uptake capacity of biomass and AC treated with acid and base. Figure 5 reveals that the adsorbed amount of both dyes at equilibrium increased with the rise in the initial dye concentration. The acid-treated AC showed the highest adsorption in comparison with the biomass sample and the base-treated AC [42]. These results were compared with previous studies, which are shown in Table 3. From the analysis of the table, it can be observed that the adsorption capacity of the prepared AC for the removal of TB and XO is comparable or superior to the values obtained in previous studies. However, the adsorbent prepared in the current study is cheaper, more eco-friendly and widely available than the aforementioned adsorbents. This increase in biomass and AC uptake capacity of the TB and XO dyes may be ascribed to π–π interactions among dye molecules and functional groups of the carbon surface since they are responsible for the mechanism of aromatic compound adsorption. Thus, increasing the initial concentration of dyes led to the increase in driving force for mass transfer, which increases the rate of dye molecule transport from the solution to the adsorbent particle surface. The increase in uptake capacity by rising dye concentrations indicated that the AC, especially the acid-treated sample, possess great potential to eliminate TB and XO from aqueous solutions [43]. 

#### 3.2.2. Adsorption Isotherms

Adsorption isotherm is significant to depict the interaction of solutes with adsorbents. The Langmuir and Freundlich isotherm models were utilized to study the relationship between the adsorbed amount of dyes and its equilibrium concentration in solutions.

##### Langmuir Isotherm Model

The Langmuir isotherm model assumes a monolayer adsorption on a surface containing a finite number of identical sites [52]. The linear form of Langmuir isotherm is shown as below:

(1) $$\frac{C_{e}}{q_{e}}=\frac{1}{q_{m}K_{L}}+\frac{C_{e}}{q_{m}}$$

where

*C_e_* = Concentration of dye molecules adsorbed at equilibrium (mg/L);
*q_e_* = Dye molecule adsorbed per unit mass of adsorbent (mg/g);
*K_L_* = Free energy of adsorption related to Langmuir isotherm constant;
*q_m_* = Maximum adsorption capacity.

**Figure 6:**
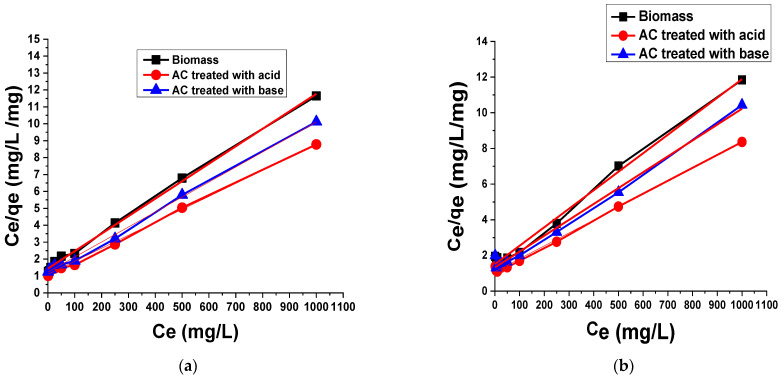
Langmuir adsorption isotherm of (**a**) TB and (**b**) XO on biomass and AC treated with acid and base.

The plot of *C_e_*/*q_e_* versus *C_e_* shows a linear relationship between the 1/*q_m_* slope and 1/*q_m_K_L_* intercept (Figure 6). The applicability of the Langmuir’s isotherm model was based on the experimental data with correlation coefficient (R^2^) of 0.99, 0.99 and 0.997 for TB adsorption on biomass, AC treated with acid, and AC treated with base, respectively, which were closed to the unity. According to this equation, the highest adsorption capacities for TB adsorption on biomass, AC treated with acid and AC treated with base were 96.81 mg/g, 130.38 mg/g, and 112.74 mg/g, respectively. The values of the Langmuir isotherm constant *K_L_* accrued from the plot were 0.0072 dm^3^/mg, 0.0079 dm^3^/mg, and 0.0072 dm^3^/mg for biomass, AC treated with acid, and AC treated with base, respectively.

The R^2^ values for the adsorption of XO on biomass, AC treated with acid and AC treated with base are 0.992, 0.997, 0.990, respectively. These values were also close to the unity. The highest adsorption capacities for the adsorption of XO on biomass, AC treated with acid and AC treated with base were 96.43 mg/g, 138.12 mg/g, and 112.36 mg/g, respectively. The *K_L_* values obtained from the plot were 0.0096 dm^3^/mg, 0.0067 dm^3^/mg, and 0.0067 dm^3^/mg, for biomass, AC treated with acid and AC treated with a base, respectively.

The dimensionless separation factor (*R_L_*), considered as an essential characteristic for the Langmuir isotherm [52], can be defined as follows:

(2) $$R_{L}=\frac{1}{1+K_{L}C_{o}}$$

The value of *R_L_* determines the type of isotherm. Specifically, $R_{L}$ > 1 means an unfavorable adsorption process, whereas favorable adsorption occurs if 0 < $R_{L}$ < 1, while values of $R_{L}$ = 1 and $R_{L}$ = 0 indicate linear and irreversible processes. The *R_L_* values were found to be 0.122, 0.126, and 0.122 in the case of TB adsorption on biomass, AC treated with acid and AC treated with a base, respectively, while in the case of XO, the values of *R_L_* were found to be 0.095, 0.131, and 0.130 for biomass, AC treated with acid and AC treated with a base, respectively. These overall discussions suggest that the current data follows the Langmuir adsorption isotherm for both dyes. Most of the previous studies for the adsorption of dyes on biomass follow the Langmuir adsorption isotherm, which indicates that the formation of dyes on the monolayer took place on the AC obtained from biomass [3].

##### Freundlich Isotherm Model

The Freundlich isotherm model assumes a heterogeneous surface on which an irregular distribution of the biosorption heat and a multilayer biosorption above the surface can be expressed [52]. The Freundlich isotherm equation is given by:

(3) $$log\frac{x}{m\;}=logK_{F}+\frac{1}{n}logC$$

**Figure 7:**
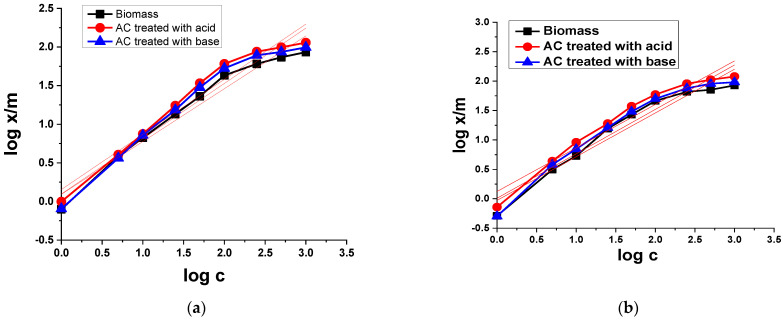
Freundlich adsorption isotherms of (**a**) TB and (**b**) XO on biomass and AC treated with acid and base.

The plot of log *x*/*m* versus *logC* shows a linear relationship with the slope of 1/*n* and intercept of *K_F_*, using the experimental data obtained, which are shown in Figure 7. The figure shows that, for the TB adsorption on biomass, AC treated with acid and AC treated with a base, the correlation coefficients (R^2^) are 0.957, 0.952, and 0.948, respectively. The values of the intercept *K_F_* obtained from the plots were 0.775, 1.246, and 0.905 for biomass, AC treated with acid and AC treated with a base, respectively. The principles of slope 1/*n* obtained from the design were 0.684, 0.714, and 0.716 for biomass, AC treated with acid and AC treated with a base, respectively.

In the case of XO, the values of correlation coefficient (R^2^) were 0.93, 0.938, and 0.930 for biomass, AC treated with acid and AC treated with a base, respectively. The values of the intercept *K_F_* resulted from the plot were 0.939, 1.332, and 1.021 for biomass, AC treated with acid and AC treated with a base, correspondingly. The principles of slope 1/*n* obtained as of the plot were 0.743, 0.740, and 0.755 for biomass, AC treated with acid and AC treated with base, respectively.

The value of *K_F_* represents the adsorption ability and 1/*n* indicates the adsorption strength. The results indicate that the adsorbent has several different types of adsorption sites. A value of n greater than 1 represents a positive adsorption situation. The value of n was found to be 1.291, 1.346, and 1.282, in the case of TB adsorption on biomass, AC treated with acid and AC treated with a base, respectively, suggesting that adsorption conditions were favorable. In the case of XO adsorption on biomass, AC treated with acid and AC treated with a base, the value of n was found to be 1.346, 1.352, and 1.324, respectively, which also indicates that the adsorption condition was favorable. However, for the Freundlich adsorption model for both dyes, the values of correlation coefficient (R^2^) were low compared to the Langmuir adsorption model.

#### 3.2.3. Contact Time

**Figure 8:**
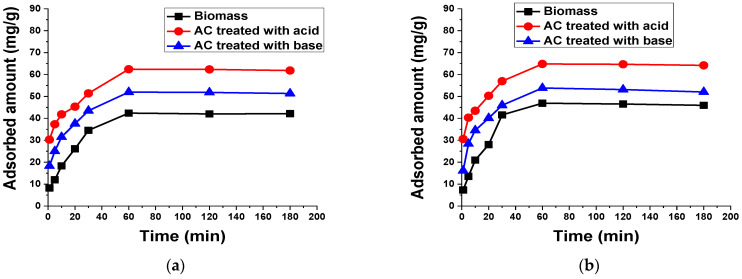
Effect of contact time on the adsorption of 50 mL (**a**) 100 mg/L TB and (**b**) 50 mg/L XO using 0.1 g biomass and AC treated with acid and base at 140 rpm and room temperature.

To assess the adsorption capacity and contact time needed by biomass and AC systems to reach equilibrium with TB and XO dyes, adsorption experiments were performed at varied contact times ranging from 1 to 180 min (Figure 8). It is clear from the figure that increasing the contact time resulted in a gradual increase in adsorption capacity, and the equilibrium was attained within the initial 60 min. These results indicate that the maximum uptake capacity is achieved within the initial 60 min. However, further increasing adsorption time had no influence on dye adsorption, which is consistent with reported results that were found in the literature for the adsorption of TB and XO dyes onto a powdered AC prepared from *Garcinia cola* nut shells [53] and coal ash [54], respectively, where an equilibrium was established within 60 min for TB adsorption and 40 min for XO adsorption. Furthermore, regarding contact time, the acid-treated AC showed a maximum adsorption capacity for both dyes.

#### 3.2.4. Kinetic Analysis

**Figure 9:**
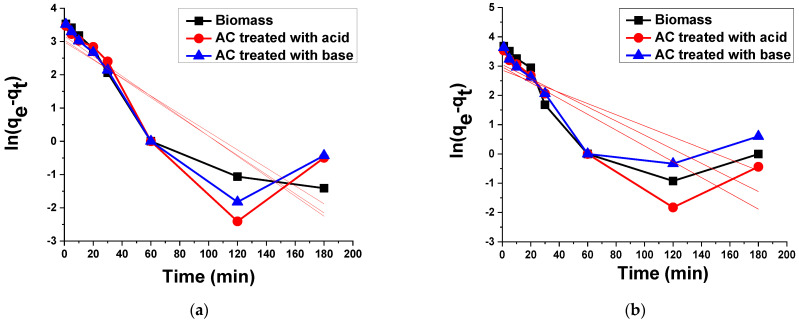
Pseudo-first order kinetic plots for the adsorption of (**a**) TB and (**b**) XO on biomass and AC treated with acid and base.

**Figure 10:**
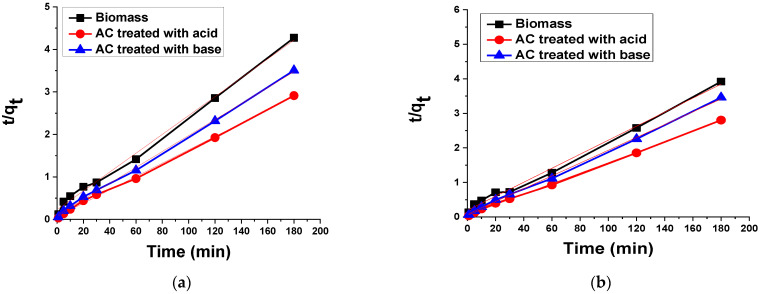
Pseudo-second order kinetic plot for the adsorption of (**a**) TB and (**b**) XO on biomass and AC treated with acid and base.

The process of removing dyes from aqueous solutions by assertiveness was studied by different kinetic models to assess the rate control process. Dynamic parameters are helpful to predict the adsorption rate, which can be utilized as important information in the design and modeling of the insurance transaction. The dynamics of dye removal have been discussed in the literature using pseudo-first and pseudo-second order dynamic models [55]. To classify and examine the mechanism of dye adsorption processes, the linearized equation of the pseudo-first order and the pseudo-second order was applied, as displayed in Figure 9 and Figure 10, respectively.

##### Pseudo-First Order Kinetic Model

This model gives information about the speed of adsorption, which is entirely depending on the capability of adsorption. The pseudo-first order kinetic model can be defined by the following equation [55]:

(4) $$ln\left(q_{e}-q_{t}\right)=lnq_{e}-k_{1}t$$

The value of pseudo-first order rate constant *k*_1_ is calculated from the intercept and slope obtained from the linear plot of *ln*(*q_e_* − *q_t_*) versus time *t*. From the plot, the values of correlation coefficient R^2^ were found to be 0.865, 0.685, and 0.709 for TB adsorption on biomass, AC treated with acid and AC treated with base, respectively. In the case of XO, the values of correlation coefficient R^2^ were found to be 0.661, 0.710, and 0.571 for biomass, AC treated with acid and AC treated with base, respectively. These results clearly indicate that the current data do not follow the pseudo-first order kinetic model due to the low correlation coefficient values.

##### Pseudo-Second Order Kinetic Model

This kinetic model is also used to define the kinetics of adsorptions with the equation below [55]:

(5) $$\frac{t}{q_{t}}=\frac{1}{k_{2}q_{e}^{2}}+\frac{t}{q_{e}}$$

where *t* is time (min), *q_t_* is the adsorbed amount of dye (mg/g) at any time (*t*), *q_e_* is the adsorbed amount of dye (mg/g) at equilibrium and *k*_2_ is the pseudo-second order rate constant.

The slope and intercept of the linear plot of *t*/*q_t_* against *t* provide the values of *q_e_* and *k*_2_. Figure 10 shows that the current data fitswell in the pseudo-second order model due to its high value of correlation coefficient. The data are also supported by previous studies in which the removal of dyes was performed with prickly pear seed cakes [56].

#### 3.2.5. pH

**Figure 11:**
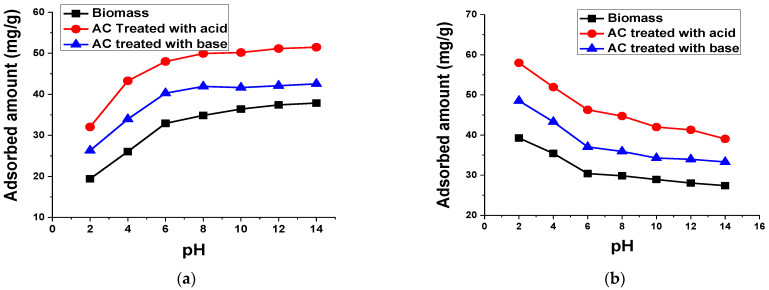
Effect of pH on the adsorption of 50 mL (**a**) 100 mg/L TB and (**b**) 50 mg/L XO using 0.1 g biomass and AC treated with acid and base for 60 min at 140 rpm and room temperature.

The influence of pH medium on the adsorption of TB and XO dyes by biomass and AC was evaluated by varying the pH of the dye samples. Figure 11 depicts the adsorption of dyes’ reliance on the solution pH. The adsorption of TB was low at a low pH on the prepared biomass and AC treated with acid and base. However, with an increase in pH, the adsorption of TB steadily increased but any further increase than pH 6 had no significant influence on the adsorption capacity of the prepared biomass and AC. These results indicate that the optimum pH for the removal of TB from wastewater is 6. On the other hand, for the anionic dye (XO), the maximum adsorption was observed at pH 2 and a rise in the pH of the dye solution decreased the adsorption capacity of the prepared biomass and AC. 

Figure 11 shows that up to pH 6, a significant decrease in pH was observed, while the further increase in pH had no significant effect on the removal capacity of XO. It is clear that, by rising the pH, the decrease in the amount of XO dye adsorption on biomass and AC occurred.

Generally, acidic dyes first dissolve and then dissociate in aqueous solutions, which results in the formation of anionic dye ions. Moreover, positive charge sites are created on the adsorbent surface in contact with water at low pH leading to a high electrostatic attraction between the anionic dye and the adsorbent of the positively charged surface. Thus, boosting the system pH causes a rise in the negatively charged sites along with a reduction in the positively charged sites, which causes the unfavorable adsorption of anionic dye because of electrostatic repulsion [57].

#### 3.2.6. Temperature

**Figure 12:**
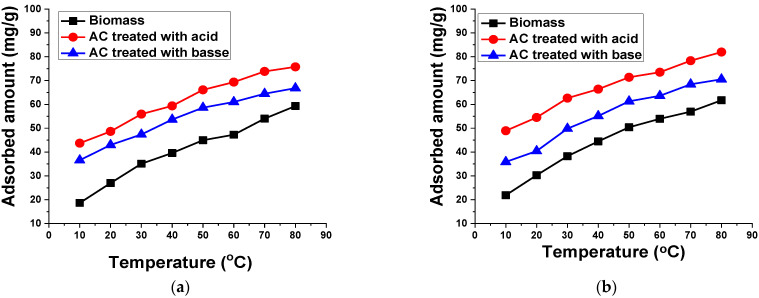
Effect of temperature on the adsorption of 50 mL (**a**) 100 mg/L TB and (**b**) 50 mg/L XO using 0.1 g biomass and AC treated with acid and base for 60 min at 140 rpm.

To investigate the effect of temperature on TB and XO dye adsorption on the prepared biomass and AC, many experiments were executed by varying the temperature from 10 °C to 80 °C, as shown in Figure 12. The adsorption of dyes is remarkably influenced by the temperature, which has an impact on the amount adsorbed on the surface of biomass and AC treated acid and base. It was found that, by rising the temperature from 10 °C to 80 °C, the uptake capacity of dyes onto the adsorbent surface was also increased. Thus, the effect of the temperature changes the adsorption efficiency of the adsorbents. From these results, it can be concluded that the adsorption of both dyes on the prepared biomass and AC treated with acid and base was chemisorption in nature. Furthermore, high temperatures can cause a stronger interaction by providing sufficient activation energy between dyes and adsorbents, which has a significant contribution in the adsorption process. Additionally, the rise in temperature of the dye solution led to the promotion of the activity of the dye molecule adsorption on the adsorbent surface, thus increasing the mass transfer through the liquid film that surrounds the adsorbent particles.

#### 3.2.7. Adsorbent Dosage

**Figure 13:**
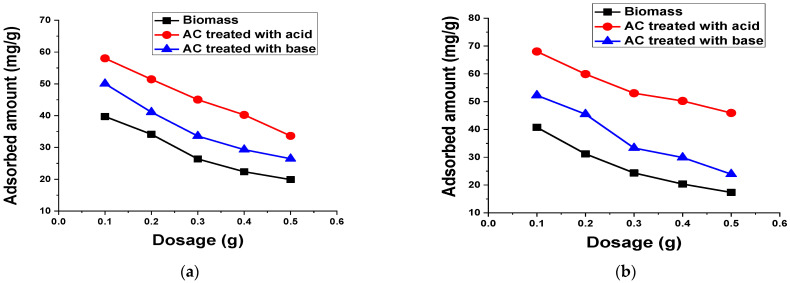
Effect of biomass and AC treated with acid and base amount on the adsorption of 50 mL (**a**) 100 mg/L TB and (**b**) 50 mg/L XO for 60 min at 140 rpm and room temperature.

To analyze the influence of the adsorbent amount on the TB and XO dyes adsorption, several experiments were carried out by changing the adsorbent concentration from 0.1 g to 0.5 g, while keeping other parameters constant (Figure 13). These findings indicate that the uptake capacity of TB and XO decreased with the increase in adsorbent amount. Such behavior is probably ascribed to the saturation of adsorption sites owing to the aggregation of adsorbent particles, which causes the decrease in the total surface area of the adsorbent and the increase in the diffusional path length [1].

#### 3.2.8. Agitation Speed

**Figure 14:**
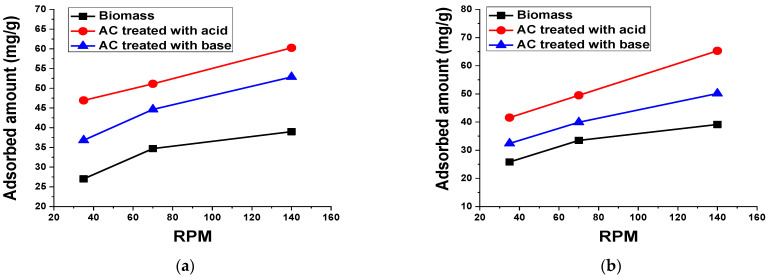
Effect of agitation speed on the adsorption of 50 mL (**a**) 100 mg/L TB and (**b**) 50 mg/L XO using 0.1 g biomass and AC treated with acid and base for 60 min at room temperature.

To evaluate the role of the agitation speed on the adsorptive elimination of dyes from aqueous solutions, different experiments were carried out by changing the agitation speed between 35 RPM and 140 RPM. The results are given in Figure 14 and indicate that the adsorption of the dyes increases significantly with the increase in agitation speed.

#### 3.2.9. Adsorbent Size

**Figure 15:**
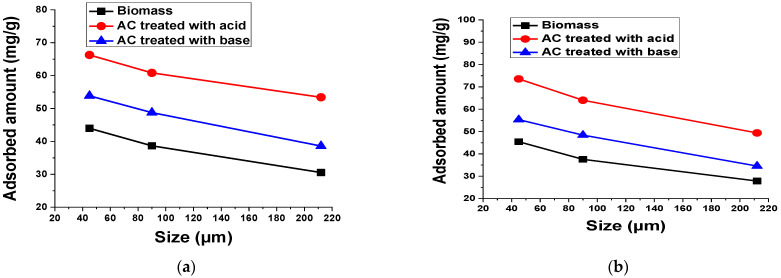
Effect of particle size for 0.1 g biomass and AC treated with acid and base on the adsorption of 50 mL (**a**) 100 mg/L TB and (**b**) 50 mg/L XO for 60 min at 140 rpm and room temperature.

The selection of particle size is the main criteria for the commercialization of the adsorbent because the small particle size causes the blockage of the fixed bed reactor, while the large particle size causes a decrease in the surface area and active sites for dye adsorption. To determine the optimum size of the adsorbent for the adsorption of the dyes, different experiments were performed by changing the particle size of adsorbent (45 µm to 212 µm) and the results obtained are shown in Figure 15. These results show that by increasing the particle size, the amount of dyes adsorbed decreased. An adsorbent with smaller particles has a large surface area, thus it has a higher adsorption capacity compared to an adsorbent with a larger particle size, which may cause a decrease in the adsorption capacity of the larger particles. Too small particle size may float on surface and not adsorb the dyes well [58].

## 4. Conclusions

In the present study, biomass and chemically synthesized AC obtained from T. F. seeds were used as bioadsorbents to remove TB and XO dyes from wastewater. Varieties of foreground techniques were used to characterize the AC prepared by using acid and base treatments. The FTIR result indicated the formation of AC from the biomass of the T. F. seeds. The formation of AC was confirmed through the disappearance of various functional groups present in biomass. The SEM results showed that the pyrolysis process produced a high porous AC in the acid-treated sample. The formation of high C/O ratio composites in the acid-treated sample proved the formation of graphitic carbon, which was confirmed by the EDX and XRD results, especially in the acid-treated sample. The effects of a variety of parameters were investigated for the removal of the TB and XO dyes on the prepared AC, such as dye concentration, contact time, pH, temperature, adsorbent amount, rpm, and absorbent size. The adsorption of TB and XO dyes increased with the rise in the initial dye concentration, and isothermal data suggested that the adsorption of both dyes followed the Langmuir isotherm model. The equilibrium time for the adsorption of the TB and XO dyes were 60 min on the biomass and synthesized AC, and the kinetic data were better matched to the pseudo-second order model. The optimal pH for TB adsorption was pH 6, while it was pH 2 in the case of XO. Due to chemisorption, increasing the temperature led to an increase in the adsorption affinity of the TB and XO dyes. The results demonstrated that the maximum adsorption was achieved at 80 °C. On the other hand, the data revealed that the uptake capacity of TB and XO decreases with the increase in adsorbent mass due to the sintering or agglomeration of the particles. The adsorption of both dyes increased with the rise in agitation speed and decreased with the increase in particle size. Thus, the current study suggests that AC prepared from T. F. seeds through an acid treatment may be considered as a perfect, eco-friendly alternative adsorbent to eliminate dyes from industrially polluted water.

## Figures and Tables

**Table 1:** BET analysis of biomass and AC treated with acid and base.

Sample	BET Surface Area (m^2^/g)	Pore Volume (cm^3^/g)	Pore Radius (Å)
Biomass	29.10	0.007	16.90
AC treated with acid	64.28	0.012	14.51
AC treated with base	43.57	0.003	16.78

**Table 2:** EDX results of biomass and AC treated with acid and base.

Biomass			Acid-Treated AC		Base-Treated AC	
Element	Weight, %	Atomic, %	Weight, %	Atomic, %	Weight, %	Atomic, %
C	58.02	64.33	76.45	83.24	61.04	56.21
O	30.97	25.78	17.44	14.26	24.52	30.08
N	9.97	9.48	0.18	0.08	12.01	12.64
Si	0.26	0.12	2.29	1.06	0.73	0.38
P	0.30	0.13	0.55	0.23	0.55	0.26
K	0.28	0.09	1.43	0.58	0.57	0.21
Ca	0.21	0.07	1.67	0.54	0.58	0.21
Total	100	100	100	100	100	100

**Table 3:** Comparison of the adsorption of XO and TB of the current results with the previous literature.

Bioadsorbent	q_max_ (mg/g)	References
	Thymol Blue (TB)	
Pomegranate peel	5.28	[44]
Garcinia cola nut shells	396.04	[45]
Hydroxyapatite powder	0.21	[46]
*Trachycarpus fortunei*	96.81 (Biomass) 130.38 (AC treated with acid) 112.74 (AC treated with base)	Current study
	Xylenol Orange (XO)	
Coal ash	0.74	[47]
Natural bauxite	2.74	[48]
Polyurethane foam	0.904	[49]
Expansion graphite	18.15	[50]
Sodium alginate graft polyhydrogel composite	312.4	[51]
*Trachycarpus fortunei*	96.43 (Biomass) 138.12 (AC treated with acid) 112.36 (AC treated with base)	Current study

## Data Availability

The data presented in this study are available on request from the corresponding author.
